# COVID-19 vaccine booster significantly decreases the risk of intensive care unit hospitalization in heart failure patients during the Omicron variant wave: A population-based study

**DOI:** 10.3389/fcvm.2022.998842

**Published:** 2022-10-20

**Authors:** Jiri Parenica, Klara Benesova, Martin Radvan, Ondrej Sanca, Jiri Hlasensky, Petr Lokaj, Tomas Ondrus, Katerina Helanova, Petr Kala, Ladislav Dusek, Jiri Jarkovsky

**Affiliations:** ^1^Internal and Cardiology Department, University Hospital Brno, Brno, Czechia; ^2^Faculty of Medicine, Masaryk University, Brno, Czechia; ^3^Department of Data Collection and Processing, Institute of Health Information and Statistics of the Czech Republic, Prague, Czechia; ^4^Faculty of Medicine, Institute of Biostatistics and Analysis, Masaryk University, Brno, Czechia

**Keywords:** COVID-19, severe course, Omicron, vaccination, heart failure

## Abstract

**Background:**

Heart failure (HF) patients are at higher risk of severe coronavirus disease 2019 (COVID-19). The Omicron variant has many novel mutations including those in the spike protein, leading to questions about vaccine effectiveness. The aim of this analysis was to evaluate the effectiveness of the COVID-19 vaccine with or without a booster (i.e., after the third dose) during the Omicron variant wave.

**Methods:**

Chronic heart failure patients in the Czech Republic were included in the analysis. COVID-19 infection was monitored from January 1st 2022 to March 31st 2022. The analysis was conducted on data collected in the National Health Information System. Vaccine effectiveness of vaccinated (with or without booster) vs. unvaccinated patients was analyzed for incidence of COVID-19, COVID-19-related hospitalizations, COVID-19 related intensive care unit admissions, and COVID-19 related mechanical ventilation/extracorporeal membrane oxygenation treatment.

**Findings:**

From a total 165,453 HF patients in the Czech Republic, 9,728 contracted COVID-19 (22.9% of them not vaccinated, 23.2% vaccinated and 53.8% vaccinated and boosted). Risk of intensive care unit (ICU) hospitalization was 7.6% in the unvaccinated group, 4.8% in the vaccinated group and 2.9% in the boosted group. The calculated effectiveness of the COVID-19 vaccine in prevention of ICU hospitalization in the vaccinated group was 41.9 and 76.6% in the boosted group.

**Interpretation:**

The results demonstrated moderate vaccine effectiveness in the prevention of severe COVID-19 in vaccinated but not boosted HF patients. Much stronger effectiveness was found in those who were vaccinated and boosted.

## Introduction

Patients with heart failure (HF), particularly older adults, are at higher risk of severe coronavirus disease 2019 (COVID-19) (1–4). Bronchopulmonary infection can lead to decompensation of chronic heart failure and congestion in the respiratory tract leaves it more vulnerable to infection (5, 6). The original severe acute respiratory syndrome coronavirus 2 (SARS-CoV-2) was first identified at the end of 2019 (7) and has been found to continuously evolve new variants which evade host immune response, increase transmission, lead to a more severe course of the disease, or lower effectiveness of treatment and vaccination. The variants of concern of SARS-CoV2 include Alpha (B.1.1.7), Beta (B.1.351), Gamma (P.1), and Delta (B.1.617.2), and each led to a new wave of the pandemic (8). Omicron (B.1.1.529) was described in November 2021 in South Africa and there was early information concerning lower virulency and increased transmission compared to earlier variants (9, 10). Omicron has many novel mutations including those in the spike protein (8, 11), leading to questions about vaccine efficacy (12, 13).

The primary aim of this analysis was to evaluate vaccine efficacy (with or without a booster dose) in preventing COVID-19 intensive care unit hospitalization in chronic heart failure patients during the Omicron variant wave.

## Methods

### SARS-CoV-2 infection

SARS-CoV-2 infection was diagnosed by real-time reverse-transcriptase-polymerase-chain-reaction (RT-PCR) assays. The Omicron variant was identified on the basis of genome sequencing, which was performed in about 1.0% of SARS-CoV-2 virus infections in the Czech Republic (14). From January 1st 2022 to March 31st 2022, a total of 1,338,068 cases of COVID-19 were diagnosed in the Czech Republic (12,503 cases per 100,000 population), the mean age was 36.5 (±19.6) years. The proportion of Omicron infections had been growing significantly since Dec 6th 2021 and prevailed during January 2022. Variant BA.1 spread first, followed by variant BA.1.1. and since mid-January, the number of patients with the BA.2 variant has been gradually increasing (14).

### Cohort construction

Chronic heart failure patients in the Czech Republic were included in the analysis. COVID-19 infection was monitored from January 1st 2022 to March 31st 2022. Hospitalization or ICU admission was considered COVID-19-related if it occurred within 14 days of a positive RT-PCR test.

The methodology for obtaining and processing data from the health information system for COVID-19 has been described elsewhere (3), briefly: the analysis was conducted on data collected in the National Health Information System (NHIS) which is managed by the Institute of Health Information and Statistics of the Czech Republic. Patients with chronic heart failure were identified using data from The National Registry of Reimbursed Health Services. The registry contains data from health insurance companies for both outpatients and inpatients including diagnoses, procedures, and medications. Data are currently available for the period 01/2010–12/2021 which is also the timeframe for the detection of patients with HF. Data on COVID-19 were extracted from the Information System of Infectious Diseases (ISID) containing, among other things, records of tests, positive cases, related hospitalizations, and vaccination status. Data in the ISID are collected in compliance with Act No. 258/2000 Coll. on Protection of Public Health, whereas data in the NHIS are collected—and linked with data from ISID—in accordance with Act No. 372/2011 Coll., on Health Services and Conditions of Their Provision. Due to this legal mandate, the retrospective analyses did not require either approval by an ethics committee or informed consent from participants.

### Statistical analysis and outcomes

Standard descriptive statistics were applied in the analysis; absolute and relative frequencies for categorical variables and means supplemented with standard deviations for continuous variables. The life-table method was applied to estimate the probability of hospital and ICU admission. Patients were followed from the day of COVID-19 diagnosis. Those without events were censored on 14th April 2022. Vaccine effectiveness of vaccinated (with or without booster) vs. unvaccinated patients was analyzed for incidence of COVID-19, COVID-19-related hospitalizations, COVID-19 related intensive care unit admissions, and COVID-19 related mechanical ventilation/extracorporeal membrane oxygenation treatment. The computation used a days at risk approach with the following formulas: a) Vaccine effectiveness of completed vaccination without booster vs. unvaccinated.


$$\begin{array}{l} 1-\frac{\overset{2022-03-31}{\underset{x=2022-01-01}{\sum }}\frac{Number\;of\;vaccinated\;without\;booster\;with\;the\;observed\;event\;on\;day\;x}{Number\;of\;vaccinated\;without\;booster\;on\;day\;x}}{\overset{2022-03-31}{\underset{y=2022-01-01}{\sum }}\frac{Number\;of\;unvaccinated\;with\;the\;observed\;event\;on\;day\;y}{Number\;of\;unvaccinated\;on\;day\;y}}, \end{array}$$

b) Vaccine effectiveness of completed vaccination with booster vs. unvaccinated


$$\begin{array}{l} 1-\frac{\overset{2022-03-31}{\underset{x=2022-01-01}{\sum }}\frac{Number\;of\;vaccinated\;with\;booster\;with\;the\;observed\;event\;on\;day\;x}{Number\;of\;vaccinated\;with\;booster\;on\;day\;x}}{\overset{2022-03-31}{\underset{y=2022-01-01}{\sum }}\frac{Number\;of\;unvaccinated\;with\;the\;observed\;event\;on\;day\;y}{Number\;of\;unvaccinated\;on\;day\;y}} \end{array}$$

Available patient characteristics (sex, age, hypertension, diabetes mellitus, malignancy in the past 5 years, history of stroke, history of renal failure, number of HF hospitalizations in the past 2 years and COVID-19 vaccine status) were used and significant predictors assessed using univariable Cox regression models. Significant factors (<0.05) were entered into a multivariable Cox regression model with backward stepwise algorithm for selection of independent predictors of COVID-19-related hospitalization and ICU admissions.

## Results

At the beginning of 2022, there were an estimated 165,453 chronic heart failure patients in the Czech Republic (point prevalence 1.546 cases per 100,000 population). Of these, 52.7% were men, with a mean age (±standard deviation) of 71.8 (±11.8) years, the mean of age of women was 78.1 (±10.8). Out of total of 165,453 patients, 45,777 (27.7%) were vaccinated with 2 doses (or 1 dose of Jansen COVID-19 vaccine) (vaccinated group), 89,127 (53.9%) patients received 3 doses (boosted group) and 30,549 (18.5%) were not vaccinated. Of a total 134,904 patients, 65.8% received the Pfizer-BioNTech vaccine, 18.4% received Oxford-AstraZeneca, 11.5% received Moderna vaccine, and 4.2% received Janssen COVID-19 vaccine. Booster doses were administered to 89,127 patients, of them 85.0% received the Pfizer-BioNTech and 15.0% received Moderna.

There were a total of 9,728 COVID-19 cases in patients with chronic heart failure, of which 54.9% were men. The unvaccinated group comprised 2,231 (22.9%) patients, vaccinated but not boosted were 2,260 (23.2%), and 5,237 (53.8%) were boosted. The baseline characteristics are shown in Table 1. All three groups were comparable in terms of age, gender, comorbidities, pharmacological treatment of chronic heart failure and number of hospitalizations for acute heart failure decompensation within the last 2 years.

**Table 1:** Comorbidities and medication of patients with chronic heart failure diagnosed with COVID-19 from January to March 2022.

	**Total**	**Not vaccinated**	**Vaccinated**	**Vaccinated + boosted**
	***N* = 9,728**	***N* = 2,231**	***N* = 2,260**	***N* = 5,237**
Men	54.9%	49.7%	53.2%	57.9%
Age (mean ± SD)	74 ± 14	73 ± 15	72 ± 15	75 ± 12
**Comorbidities**				
Hypertension	83.9%	79.9%	83.4%	85.8%
Diabetes mellitus	36.1%	34.7%	35.2%	37.1%
Malignancy in the past 5 years	7.4%	6.5%	6.2%	8.2%
History of stroke	8.7%	9.3%	8.1%	8.8%
History of renal failure	6.8%	4.3%	7.2%	7.7%
**No. of HF hospitalizations in the past 2 years**				
0	75.9%	75.2%	71.6%	78.0%
1	17.2%	17.7%	19.4%	16.0%
≥2	7.0%	7.0%	9.0%	6.1%
**Medication**				
ACEI/ARB/ARNI	72.6%	69.3%	74.8%	73.0%
Spironolactone/ eplerenone	48.8%	48.1%	49.0%	49.0%
Furosemide	72.0%	71.2%	72.3%	72.2%
Digoxin	10.6%	11.8%	9.6%	10.5%
Betablockers	76.8%	73.2%	76.9%	78.4%
Dapagliflozin/ empagliflozin	3.8%	4.0%	3.6%	3.8%
No. of drugs (mean ± SD)	2.8 ± 1.2	2.8 ± 1.3	2.9 ± 1.2	2.9 ± 1.2

ACEI, angiotensin-converting enzyme inhibitors; ARB, angiotensin II receptor blockers; ARNI, angiotensin receptor-neprilysin inhibitor.

**Figure 1:**
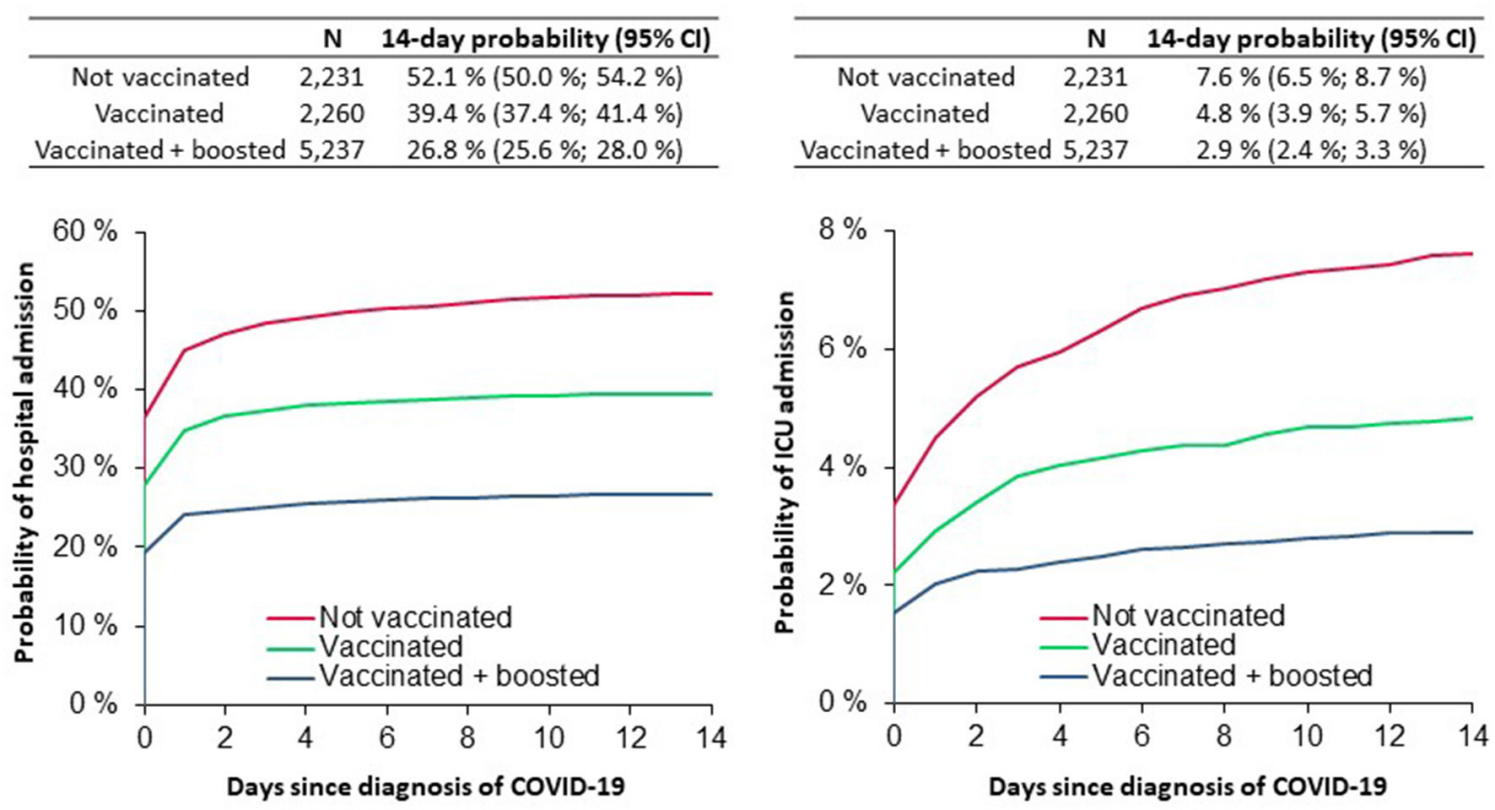
The probability of hospital/ICU admission in heart failure patients during the Omicron variant wave A.

The risk of hospitalization within 14 days of a COVID-19 diagnosis was 52.1% with 95% confidence interval (CI) (50.0–54.2%) in the unvaccinated group, 39.4% (37.4–41.4%) in the vaccinated group, and 26.8% (25.6–28.0%) in the boosted group. Most hospitalizations occurred within 2 days of a

positive test. The risk of intensive care was 7.6% (6.5–8.7%) in the unvaccinated group, 4.8% (3.9–5.7%) in the

vaccinated group and 2.9% (2.4–3.3%) in the boosted group (Figure 1).

The calculated vaccine effectiveness of 5.7% for COVID-19 positivity demonstrates the small benefit of completed vaccination compared with the unvaccinated group. Vaccination efficacy against intensive care unit admissions was 41.9%. The effectiveness in the boosted population was 76.6% for intensive care unit admissions and 79.2% for mechanical ventilation/extracorporeal membrane oxygenation treatment (Table 2).

**Table 2:** Vaccine effectiveness in heart failure patients within the period Jan–Mar 2022.

	**Effectiveness of completed vaccination without booster**	**Effectiveness of completed vaccination with booster**
**Population**		
Vaccine effectiveness for positivity	5.7%	33.6%
Vaccine effectiveness against hospitalization	26.4%	66.2%
Vaccine effectiveness against ICU hospitalization	41.9%	76.6%
Vaccine effectiveness against ventilation/ECMO	48.3%	79.2%

Using univariable and multivariable Cox regression analysis, models for prediction of COVID-19 ICU admission in chronic heart failure patients diagnosed with COVID-19 were calculated (Supplementary Table 1). Vaccination and vaccination with booster dose were independent predictors of COVID-19 related ICU admissions. Hazard ratio for ICU admission was 0.61 (CI 0.48–0.77; *p* < 0.001) for vaccinated group and 0.36 (CI 0.29–0.44; *p* < 0.001) for boosted group.

## Discussion

The present analysis demonstrates the protective effect of vaccination against severe COVID-19 in a large, real-world population of 165,453 chronic heart failure patients. There was a clear reduction in-hospital intensive care for heart failure patients with history of vaccination, which was more pronounced in those with a booster dose. There was a demonstrated decreased rate of any hospitalization due to COVID-19 in the vaccinated group. These results confirm the efficacy of vaccination against Omicron variants.

The safety and efficacy of COVID-19 vaccines approved in the Czech Republic- Pfizer-BioNTech COVID-19 vaccine (15), Moderna (16), Janssen (17), Oxford-AstraZeneca (18), and Nuvaxovid (19) were confirmed in randomized, placebo-controlled trials. Vaccine effectiveness in preventing severe COVID-19 was reported to be in the range of 85.4–100%. It should be noted that the number of severe cases in the study ranged up to a maximum of ten patients and the trials were conducted before widespread circulation of the SARS-CoV-2 Delta variant and Omicron variant.

According to the presented results vaccine effectiveness was slightly lower compared to published results: vaccine effectiveness against the most severe outcome of hospitalizations, including invasive mechanical ventilation or death, was reported as 90%. Vaccine effectiveness was 94% after three doses during the Omicron wave (20).

It is well-documented that patients with chronic heart failure, primarily characterized by advanced age, advanced heart failure and the presence of comorbidities, have an increased risk of hospitalization and severe disease due to COVID-19 (1–4, 21). In our analysis, we expressed the severity of heart failure by the number of drugs for the treatment of heart failure (22) and by the number of heart failure hospitalizations in the last 2 years (23). It is known that infection is a common precipitating factor in acute decompensation of heart failure (24). Acute myocardial injury, myocardial infarction, myocarditis, respiratory failure, secondary bacterial infection, or renal injury could be other reasons leading to acute decompensation of chronic heart failure and worsening prognosis of these patients during COVID-19 (25).

The main limitations of the study are non-randomized design covering potential differences between the unvaccinated and vaccinated group and the fact that it does not distinguish between hospitalizations due to COVID-19 or those where COVID-19 was not the main reason for the hospital stay. Several studies have shown that vaccine effectiveness against hospitalization appears to be lower for Omicron variant compared to previous variant (26, 27). One reason could be because with Omicron incidences have become much higher than experienced ever before and there are more “with but not because of COVID-19” hospitalizations and consequently lower vaccine effectiveness. Vaccine effectiveness estimates improve when definitions of endpoints are chosen more specific to severe respiratory disease (28, 29).

Theis study had number of other limitations: the analysis did not consider previous COVID-19 infections and time from the vaccination. We cannot rule out a small number of patients who may have developed COVID-19 and not been tested using RT-PCR testing. We are not able to clearly distinguish patients with preserved, mid-range and reduced ejection fraction. Body mass index as important characteristic of heart failure population (30) and negative prognostic factor of COVID-19 (31) was not known and not included in the analysis. It was also hypothesized that physical activity and air pollution could play a role in the spread of the virus (32, 33), our data do not allow us identifying patients who live in geographic areas with increased air pollution.

## Conclusions

Heart failure patients represent a group at high risk of severe COVID-19. Despite several study limitation our study demonstrates that approved COVID-19 vaccines remained effective against COVID-19- associated ICU hospitalization among a large population of patients with heart failure during the SARS-CoV-2 Omicron wave. Booster doses significantly increased the protection effect.

The results continue to highlight the urgent need to educate patients with chronic heart failure about the benefits of vaccination and the booster dose with existing approved vaccines, even during the relaxation of measures against coronavirus.

## Research in context

### Evidence before this study

Heart failure patients are at higher risk of severe COVID-19. The Omicron variant has prevailed since January 2022.

### Added value of this study

Vaccine effectiveness in preventing severe COVID-19 with admission to the ICU was 41.9% in heart failure patients. The booster dose significantly increased the protective effect of vaccination and lead to vaccine effectiveness of 66.2% in preventing hospitalization and 76.6% in ICU admission.

### Implications of all the available evidence

It is important that heart failure patients be educated about the protective effect of vaccination and the booster dose which brings strong protection against severe COVID-19 with the Omicron variant.

### Data sharing

The anonymized data is available upon reasonable request. The data are deidentified participant data, and available from the author JJ (jiri.jarkovsky@uzis.cz). The reuse of the data subset is permitted only for revalidation of the results.

## Data availability statement

The analysis was performed with data from the National Health Information System (NHIS), which was supplemented with data from the Information System of Infectious Diseases (ISID). Data in the ISID are collected in compliance with Act No. 258/2000 Coll. on Protection of Public Health, data in the NHIS are collected-and linked with data from ISID-in accordance with Act No. 372/2011 Coll., on Health Services and Conditions of Their Provision. The anonymized data is available upon reasonable request. The data are deidentified participant data, and available from the author JJ (jiri.jarkovsky@uzis.cz). The reuse of the data subset is permitted only for revalidation of the results.

## Ethics statement

Ethical review and approval was not required for the study on human participants in accordance with the local legislation and institutional requirements. Written informed consent for participation was not required for this study in accordance with the national legislation and the institutional requirements.

## Author contributions

JP: methodology, resources, validation, writing-original draft, visualization, formal analysis, project administration, and funding acquisition. KB: visualization and formal analysis. MR, JH, OS, and KH: visualization, formal analysis, and project administration. PL and TO: writing-original draft, visualization, and formal analysis. PK: project administration and funding acquisition. LD: project administration, funding acquisition, and formal analysis. JJ: project administration and supervision. All authors contributed to the article and approved the submitted version.

## Funding

This research was supported by a grant from the Czech Republic Operational Program eHealth and Rare Disease CZ.03.4.74/0.0/0.0/15_025/ and by a grant from the Ministry of Health of the Czech Republic—conceptual development of research organization (FNBr, 65269705; funding was given to University Hospital Brno).

## Conflict of interest

The authors declare that the research was conducted in the absence of any commercial or financial relationships that could be construed as a potential conflict of interest.

## Publisher's note

All claims expressed in this article are solely those of the authors and do not necessarily represent those of their affiliated organizations, or those of the publisher, the editors and the reviewers. Any product that may be evaluated in this article, or claim that may be made by its manufacturer, is not guaranteed or endorsed by the publisher.
