# Supplementary material for: COVID-19 vaccine booster significantly decreases the risk of intensive care unit hospitalization in heart failure patients during the Omicron variant wave: A population-based study
Source: Front Cardiovasc Med. 2022 Oct 20;9:998842. doi: 10.3389/fcvm.2022.998842 (PMC9631812; doi:10.3389/fcvm.2022.998842)
Supplement: Supplementary file 1 [file Table_1.docx]

**Supplemental Table 1** Univariable and multivariable Cox regression models for prediction of COVID-19 ICU admission in patients with chronic heart failure diagnosed with COVID-19 between January and March 2022

| **Predictors** |  | **Univariable models** | | **Multivariable model** | |
| --- | --- | --- | --- | --- | --- |
|  |  | **HR (95% CI)** | **P-value** | **HR (95% CI)** | **P-value** |
| Sex | Women (ref. men) | 0.77 (0.64–0.93) | **0.007** | 0.73 (0.60–0.88) | **0.001** |
| Age | 10-year increase | 1.01 (0.94–1.08) | 0.826 | - | - |
| Hypertension | Yes (ref. no) | 1.23 (0.93–1.61) | 0.141 | - | - |
| Diabetes mellitus | Yes (ref. no) | 1.45 (1.20–1.75) | **< 0.001** | 1.43 (1.18–1.73) | **< 0.001** |
| Malignancy in the past 5 years | Yes (ref. no) | 1.36 (0.99–1.86) | 0.057 | - | - |
| History of stroke | Yes (ref. no) | 1.01 (0.72–1.40) | 0.967 | - | - |
| History of renal failure | Yes (ref. no) | 1.47 (1.08–2.02) | **0.016** | 1.48 (1.08–2.04) | **0.015** |
| No. of HF hospitalizations in the past 2 years | 1 (ref. 0) | 1.51 (1.20–1.89) | **< 0.001** | 1.46 (1.16–1.83) | **0.001** |
|  | ≥ 2 (ref. 0) | 1.79 (1.32–2.43) | **< 0.001** | 1.65 (1.21–2.24) | **0.001** |
| COVID-19 vaccine status | Vaccinated (ref. not vaccinated) | 0.64 (0.50–0.81) | **< 0.001** | 0.61 (0.48–0.77) | **< 0.001** |
|  | Vaccinated + booster (ref. not vaccinated) | 0.37 (0.30–0.46) | **< 0.001** | 0.36 (0.29–0.44) | **< 0.001** |

CI, confidence interval; HR, hazard ratio; Ref., reference category.
